# Personality drives activity and space use in a mammalian herbivore

**DOI:** 10.1186/s40462-022-00333-6

**Published:** 2022-08-13

**Authors:** Jonas Stiegler, Alisa Lins, Melanie Dammhahn, Stephanie Kramer-Schadt, Sylvia Ortmann, Niels Blaum

**Affiliations:** 1 Institute of Biochemistry and Biology, Plant Ecology and Nature Conservation, University of Potsdam, Potsdam, Germany; 2 Department for Behavioral Biology, University of Münster, Münster, Germany; 3 Leibniz Institute for Zoo and Wildlife Research (IZW), Berlin, Germany; 4 Institute of Ecology, Technische Universität Berlin, Berlin, Germany

**Keywords:** Animal personality, Movement ecology, Inter-individual differences, ODBA, Energy expenditure, European hare

## Abstract

**Background:**

Animal personality has emerged as a key concept in behavioral ecology. While many studies have demonstrated the influence of personality traits on behavioral patterns, its quantification, especially in wild animal populations, remains a challenge. Only a few studies have established a link between personality and recurring movements within home ranges, although these small-scale movements are of key importance for identifying ecological interactions and forming individual niches. In this regard, differences in space use among individuals might reflect different exploration styles between behavioral types along the shy-bold continuum.

**Methods:**

We assessed among-individual differences in behavior in the European hare (*Lepus europaeus*), a characteristic mammalian herbivore in agricultural landscapes using a standardized box emergence test for captive and wild hares. We determined an individuals’ degree of boldness by measuring the latencies of behavioral responses in repeated emergence tests in captivity. During capture events of wild hares, we conducted a single emergence test and recorded behavioral responses proven to be stable over time in captive hares. Applying repeated novel environment tests in a near-natural enclosure, we further quantified aspects of exploration and activity in captive hares. Finally, we investigated whether and how this among-individual behavioral variation is related to general activity and space use in a wild hare population. Wild and captive hares were treated similarly and GPS-collared with internal accelerometers prior to release to the wild or the outdoor enclosure, respectively. General activity was quantified as overall dynamic body acceleration (ODBA) obtained from accelerometers. Finally, we tested whether boldness explained variation in (i) ODBA in both settings and (ii) variation in home ranges and core areas across different time scales of GPS-collared hares in a wild population.

**Results:**

We found three behavioral responses to be consistent over time in captive hares. ODBA was positively related to boldness (i.e., short latencies to make first contact with the new environment) in both captive and wild hares. Space use in wild hares also varied with boldness, with shy individuals having smaller core areas and larger home ranges than bold conspecifics (yet in some of the parameter space, this association was just marginally significant).

**Conclusions:**

Against our prediction, shy individuals occupied relatively large home ranges but with small core areas. We suggest that this space use pattern is due to them avoiding risky, and energy-demanding competition for valuable resources. Carefully validated, activity measurements (ODBA) from accelerometers provide a valuable tool to quantify aspects of animal personality along the shy-bold continuum remotely. Without directly observing—and possibly disturbing—focal individuals, this approach allows measuring variability in animal personality, especially in species that are difficult to assess with experiments. Considering that accelerometers are often already built into GPS units, we recommend activating them at least during the initial days of tracking to estimate individual variation in general activity and, if possible, match them with a simple novelty experiment. Furthermore, information on individual behavioral types will help to facilitate mechanistic understanding of processes that drive spatial and ecological dynamics in heterogeneous landscapes.

**Supplementary Information:**

The online version contains supplementary material available at 10.1186/s40462-022-00333-6.

## Introduction

In the paradigm of movement ecology, an animals' space use is explained as a consequence of the influence of external factors on three movement processes: internal state, navigation, and motion capacity [1]. An increasing number of studies show high among-individual differences in movement characteristics that cannot be explained by the paradigm alone [2, 3]. Spiegel et al. [2] refined this paradigm by including personality-dependent spatial ecology and suggested that among-individual differences in behavioral types are a predictor for space use. Consistent among-individual variation of behavior over time is referred to as animal personality [4], a key concept in behavioral ecology [3, 5–7].

Although animal personality is widely recognized as a critical intrinsic component of behavior, its quantification under field conditions remains a challenge for two main reasons. First, extensive species-specific standardized tests need to be developed and performed repeatedly to measure consistency in among-individual differences in behavior [8]. Under such test conditions, restraining individuals could interfere with the procedures and objectives of the intended field study, and direct observation of individuals could be impossible. Second, observed among-individual differences in behavior need to be ecologically validated [4]. In most field studies, this would result in a prolonged handling time of individuals, which contrasts with minimizing effects on trapped animals or maximizing sample size [9].

Réale et al. [4] summarized the fundamental personality traits as activity, boldness, exploration behavior, aggressiveness, and sociability. While most of them are difficult to quantify in the wild, the personality trait activity—defined as an individual's general level of activity—can be recorded using accelerometers. Hence, such derived measurements may allow for a remote classification of behavioral types. The main aim of this study was to assess whether and how aspects of animal personality (quantified in standardized and repeated emergence and novel environment tests) and general activity (quantified via accelerometers) are related and whether they are correlated with space use. Moreover, our novel approach shows a feasible way to combine experiments in a controlled environment with ones in the wild. Thus, we contribute to answering a fundamental question in personality research and movement ecology, namely how behavioral traits covary with movement strategies in the wild [10].

Previous research highlights that among-individual differences in behavior, i.e., animal personality, are a key aspect of variation in "internal states" underlying movement and space use [11] with individuals varying consistently in how, where, and when they move [2, 12]. Variation, particularly along the shy-bold continuum [13, 14], is suggested to affect crucial ecological processes, e.g., predation rates [15] or population structure [12, 16], and to generate spatio-temporal variability that influences individuals' interactions with biotic and abiotic factors [5, 17–20]. For example, boldness and exploration have been shown to correlate with variation in foraging patterns [21, 22] or habitat use [2, 23]. However, only a few studies focused on local movement types [but see: 32–34], even though small-scale movements are of crucial importance for ecological interactions [24–28], the formation of individual niches [29, 30], and hence the community dynamics and species coexistence [19, 31–35]. In addition to movement data derived from GPS devices, tri-axial accelerometers measure static and dynamic acceleration (i.e., the animals' movement) in three dimensions [36]. These measurements can be used to remotely identify acceleration patterns and assign them to defined behaviors [37, 38] or calculate proxy values for activity levels [39–42]. One well-established example is the overall dynamic body acceleration (ODBA), allowing us to estimate a free-ranging animals' activity-related energy expenditure after careful validation [39, 43]. Accelerometers are increasingly implemented in studies of animal behavior, ecology, or physiology [44–54] and may be a promising tool to assess animal personalities remotely [22].

In the present study, we experimentally identify and link behavioral types of a mammalian herbivore (*Lepus europeaus*) to their general activity recorded by accelerometers. After relating the individuals’ degree of boldness to its’ activity in both captive and wild hares, we ultimately test for their association with space use in a wild population. Recent studies on space use of the European hare found high among-individual variation in movement patterns that remained hitherto largely unexplained [55, 56]. Here, we present a 3-step approach in which we (i) quantify and test the repeatability of among-individual differences in behavior along the bold-shy continuum of captive hares with repeated standardized emergence and novel environment tests in an open-field arena, (ii) link the degree of boldness to a captive individual's general activity in the arena derived from accelerometers and assessed via ODBA, and (iii) investigate the association of wild hares activity and space use with their degree of boldness, estimated from behavioral responses along the shy-bold continuum, proven to be temporarily stable in captive hares. In this final step, we explore the relation of repeatable metrics of behavior linked to animal personality, with space use in a disturbance-mediated agricultural landscape described by Ullmann et al. [55, 56]. More specifically, we relate home range size and the area size to an individual’s position along the shy-bold continuum.

We hypothesize that similar to findings in small [33, 57, 58] and medium sized mammals [59] general activity (i.e., ODBA) and boldness are positively linked in hares. Further, we expect that boldness predicts space use with bolder individuals allocating both a larger home range and a larger core area.

## Methods

### Step 1-animal personality tests in captive hares

Captive European hares were studied at the field station of the Leibniz Institute for Zoo and Wildlife Research (IZW), located about 40 km north of Berlin (Brandenburg, Germany, 52°51′06.5′′N, 13°54′57.2′′E; WGS84). There, hares are kept and bred for reproduction studies [e.g., 79]. All individuals used in this study are housed singly in small cages of 2 m^2^ or arenas with concrete floors (5 m x 10 m, where two individuals share the enclosure).

In July and August 2019 and 2020, we conducted repeated novel environment tests with 14 captive hares in an enclosure, that is an open field arena (8 m × 27 m) surrounded by a transparent metal mesh fence with a height of 180 cm. The area was freshly mowed and equipped with a 2 m^2^ housing box as a familiar retreat site, a small shelter on the opposite side, and two troughs containing food and water ad libitum.

The individual was weighed and transferred to the new enclosure in a wooden box (60 cm x 25 cm x 30 cm). This box was placed in the front right corner inside the enclosure, carefully opened, and remained untouched. Each hare was individually tested while remaining in the enclosure for three consecutive days. In total, we tested 14 individuals in the novel environment test; 12 of them provided GPS/ACC recordings. All experiments were continuously videotaped with a GPS-synced digital camera [60] during periods of sufficient daylight (i.e., 06:00–22:00). Repetition trials were conducted after two weeks. Latencies of specific behaviors (Latency look, Latency leave, and Delta look-leave; definitions in Table 1) were determined from video recordings by one observer (AL).

**Table 1:** Definitions, min–max range, and units of behaviors observed during novel environment tests with captive European hares (*Lepus europaeus*) in an open field arena and on the release of captured free-ranging hares. ODBA data are based on acceleration loggers applied in collars

Behavior	Definition	Unit	Range†	Location*
Latency look°	Latency until the eyes were above the edge of the box for the first time	$\left[s\right]$	1–3,951	Both
Latency leave°	Latency until the hare was outside the box with its full body for the first time	$\left[s\right]$	1–3,961	Both
Delta look-leave°	Delta between Latency look & Latency leave	$\left[s\right]$	0–3482	Both
Exploring the first 3 m°	Latency from Latency leave until the hare crossed the first 3 m of the enclosure with its entire body	$\left[s\right]$	40–7,817	Enclosure
Exploring the enclosure°	Latency from Latency leave until the hare reached the opposite end of the enclosure with its full body (27 m)	$\left[s\right]$	66–12,000	Enclosure
3-day activity	Mean ODBA value for three successive days, calculated after the release of the individual (72 h)	$\left[\frac{m}{{s}^{2}}\right]$	0.10–0.51	Both
10-day activity	Mean ODBA value for 10 successive days, calculated after the release of the individual. (240 h)	$\left[\frac{m}{{s}^{2}}\right]$	0.16–0.30	Field
20-day activity	Mean ODBA value for 20 successive days, calculated after the release of the individual (480 h)	$\left[\frac{m}{{s}^{2}}\right]$	0.18–0.31	Field
30-day activity	Mean ODBA value for 30 successive days, calculated after the release of the individual (720 h)	$\left[\frac{m}{{s}^{2}}\right]$	0.17–030	Field

*Behaviors were recorded at the enclosure site, in the field, or at both locations

°Behaviors were recorded for both primary and repetition trials of the captive-bred hares

†12,000 was used as a maximum value

### Step 2-ODBA in captive hares

Prior to testing, each individual was collared with a GPS device with an internal 3-axial accelerometer (ACC) weighing 69 g (< 2% of a hares' body mass, model 1AA, e-obs GmbH). Acceleration was recorded at 33 Hz (byte count 495) every 2 min and ODBA values were calculated with the R package moveACC [61] as $ODBA=\left|{A}_{x}\right|+\left|{A}_{y}\right|+|{A}_{z}|$, where A_x_, A_y_, and A_z_ are the derived dynamic accelerations corresponding to the three perpendicular axes of the sensor [40] (Tab. 1).

### Step 3—ODBA and home range of free-ranging wild hares

The field study site was located in an agricultural landscape 100 km northeast of Berlin (53°21′22.8′′ N, 13°48′03.0′′ E; WGS84) within the "AgroScapeLab Quillow" catchment, the research platform of the Leibniz Centre for Agricultural Landscape Research (ZALF) and the BioMove research training group. The climate is described as continental/Atlantic transition with long dry phases in spring and cold winters. The mean annual precipitation is 486 mm and the mean annual air temperature is 8.4 °C. The landscape is dominated by loamy soils and intensive cultivation of winter cereals, rape, and maize. The field sizes are on average 27.5 ± 1.1 ha [29, 56].

Wild hares (n = 14, Additional file 1: Table S1) were trapped by chasing them into woolen nets [for details, see 76,83], weighed, sexed, and equipped with GPS/ACC collars (model 1AA, e-obs GmbH [62]). While all hares were adults, the exact age was not determined to reduce the duration of time the animals were exposed to handling stress. An acceleration informed GPS frequency was programmed as follows: GPS locations were recorded every 4 min during normal and high activity. When no activity was recorded, GPS fixes were logged every hour [56]. Tracking data were stored in the Movebank data repository [63]. Acceleration recordings were programmed as for the captive hares. After collar fitting, hares were moved inside a wooden transport box (60 cm × 25 cm × 30 cm) to an open area in the field. At the release point, the top plate of the box was opened, and latencies for looking out of the box and leaving the box were recorded according to the novel environment test (Table 1). From untangling the hare from the net until releasing it from the box, the handling procedure lasted between 30 to 45 min. Remote data download was triggered whenever an individual was within range of a base station (model basis 5, e-obs GmbH) deployed near the trap location. Hares were tracked for a varying duration, depending on the coverage of the receiver antennas and the battery life of the collar (Additional file 1: Table S1).

### Statistical analyses

First, we estimated adjusted repeatability of behaviors in captive hares (Latency look, Latency leave, Delta look-leave, Exploring first 3 m, Exploring enclosure; Table 1) using linear mixed effect models and bootstrapping (number of parametric bootstraps for interval estimation: 10000; number of permutations to calculate asymptotic p-values: 10,000; *p*-values shown refer to repeatability) with the individual as a random factor and adjusting for housing type as a fixed effect with the R package rptR [64, 65].

Second, we tested whether repeatable among-individual differences in behavior explain variation in recorded ODBA. As "Delta look leave" (Table 1) is derived from both Latency leave and Latency look and the latter correlated among each other (Pearson correlation coefficient > 0.7), we considered Latency look and Latency leave in separate models for subsequent analyses. We calculated a linear mixed effect model with the latency as a response variable, housing type and number of the respective trial (i.e., first or second measure of the latency) as fixed effects and individual as a random effect. Following Hertel et al. [66], we derived the best linear unbiased predictor (BLUP) by extracting the conditional modes of the random effect (individual) from the fitted model. Then, we calculated a generalized linear model with activity (mean ODBA over the 3 days in the exclosure) as a response variable, Gamma error distribution and predictor variables BLUP, body mass and housing type. Then, we related latencies of wild hares to the mean ODBA during the first 3, 10, 20 and 30 days of tracking per individual (Additional file 1: Table S1).

Body mass was included in both models as a fixed effect. As captive hares experienced two types of housing conditions (small cages of 2 m^2^ with one individual; small arenas of 50 m^2^with two individuals), we additionally included housing as a fixed effect in the models. Subsequently, for captive hares we performed a step-wise backward model selection based on the information criterion Akaike (AICc, corrected for small sample size) using the dredge function implemented in the R package MuMIn [67]. Following the studies of Anderson and Burnham [68] and Pinheiro and Bates [69], we selected the model with the highest Akaike score (lowest AICc value) to best explain our data. All models within 2 AICc units were considered as competing models (Additional file 1: Tables S2 and S3).

Third, we tested if among-individual differences in behavior, i.e., boldness expressed as short latencies, predict space use in wild hares. We first calculated the cumulative home range sizes of consecutive days (day 1 to 32, Additional file 1: Fig. S1) to assess how many tracking days are needed to reach home range size saturation. After visual inspection (Additional file 1: Fig. S1), we decided to calculate home range sizes of the initial 20 and 30 (n = 12, Additional file 1: Table S1) days after releasing the captured hares. Then, we calculated home range sizes based on 95% (home range) and 50% (core area) of the kernel utilization distribution while considering autocorrelation for continuous time (akde; R package move [70] and ctmm [71]). Finally, we related the logarithmized home range and core area sizes to the predictor variables “body mass” and Latency look or “Latency leave” with Gaussian error distribution (quantile residuals were checked using the DHARMa package [72]). Due to the low sample size of captive hares (repeatability: n = 14, 4 females, 10 males; ODBA: n = 12, 4 females, 8 males) and wild hares (see Additional file 1: Table S1), we did not further analyze the effects of sex. All analyses were performed in R version 4.0.2 [57] and R Studio version 1.2.5019 [59].

## Results

### Among-individual differences in behavior and their linkage to acceleration data

**Fig. 1:**
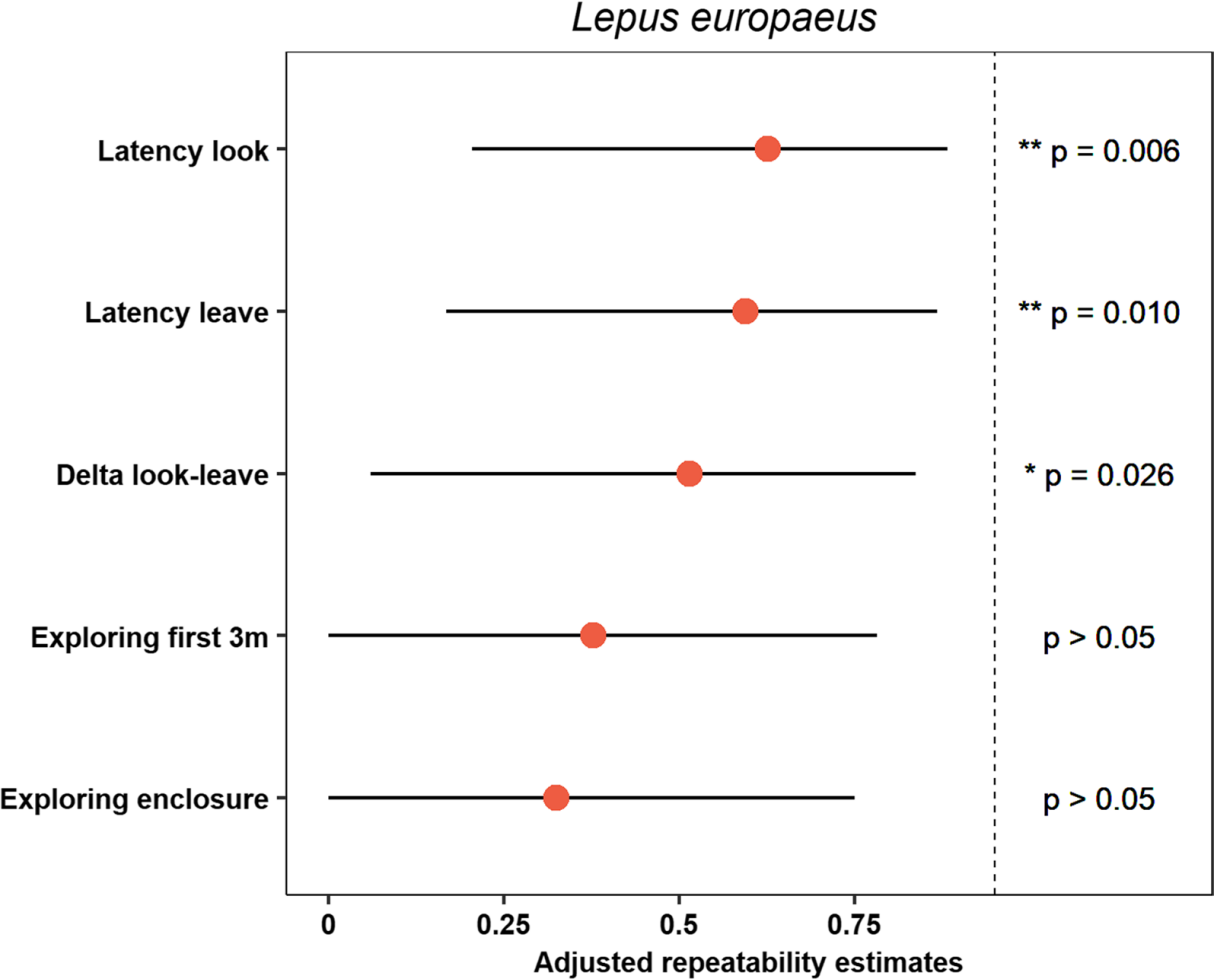
Adjusted repeatability (i.e., fixed effect for enclosure type, 10 hares were kept in cages, 4 hares were kept in arenas) of behavioral variables quantified in repeated novel environment tests of 14 captive European hares (*Lepus europaeus*) in an open field arena. Latency look: p (LRT) = 0.006, p (permutation) = 0.009; Latency leave: p (LRT) = 0.010, p (permutation) = 0.014; Delta look-leave: p (LRT) = 0.026, p (permutation) = 0.030; Exploring first 3 m: p (LRT) = 0.093, p (permutation) = 0.124; Exploring enclosure: p (LRT) = 0.135, p (permutation) = 0.183. Shown are repeatability estimates (red dots) and their 95% confidence intervals (lines) estimated via parametric bootstraps (n = 10,000 simulations); p-values are based on permutations (n = 10,000)

We found temporal consistency in three of the five behavioral variables (n = 14, all R ≥ 0.5, all *p* < 0.05): Latency look (R = 0.62 ± 0.18, *p* = 0.014), Latency leave (R = 0.59 ± 0.18, *p* = 0.021) and Delta look-leave (R = 0.49 ± 0.20, *p* = 0.021). All other behavioral variables were not repeatable over time (Table 1, Fig. 1).

**Table 2:** Model results: activity measurements in relation to the BLUP (derived from Latency look ~ housing type + number of trial + (1|individual) and Latency leave ~ housing type + number of trial + (1|individual) for captive hares, and to Latency look and Latency leave for wild hares †

Hares	Activity period	Latency look					Latency leave			
		Coefficients	Estimate	Std. error	t value	Pr( >|t|)	Estimate	Std. error	t value	Pr( >|t|)
Captive	3 days	(Intercept)	4.796	0.476	10.071	< 0.001	3.804	0.383	9.938	< 0.001
		BLUP	0.056	0.014	4.13	0.003	0.097	0.042	2.292	0.045
		Housing	− 1.099	0.535	− 2.053	0.07	−	−	−	−
Wild	3 days	(Intercept)	0.363	0.172	2.106	0.059	0.341	0.162	2.107	0.059
		Latency	− 0.002	0.001	− 2.564	0.026	− 0.002	0.001	− 2.717	0.020
		Mass	− 0.024	0.038	− 0.619	0.548	− 0.016	0.036	− 0.447	0.664
Wild	10 days	(Intercept)	0.443	0.135	3.286	0.008	0.410	0.125	3.289	0.008
		Latency	− 0.002	0.001	− 3.276	0.008	− 0.002	0.000	− 3.492	0.006
		Mass	− 0.039	0.030	− 1.307	0.221	− 0.029	0.028	− 1.044	0.321
Wild	20 days	(Intercept)	0.515	0.128	4.028	0.003	0.471	0.120	3.939	0.003
		Latency	− 0.002	0.001	− 3.779	0.004	− 0.002	0.000	− 3.884	0.004
		Mass	− 0.051	0.028	− 1.795	0.106	− 0.038	0.027	− 1.416	0.191
Wild	30 days	(Intercept)	0.477	0.141	3.397	0.011	0.371	0.138	2.676	0.032
		Latency	− 0.002	0.000	− 4.714	0.002	− 0.002	0.000	− 4.613	0.002
		Mass	− 0.040	0.030	− 1.302	0.234	− 0.013	0.031	− 0.408	0.695

†For our dependent variable, the mean ODBA, we assumed a Gamma error distribution

**Fig. 2:**
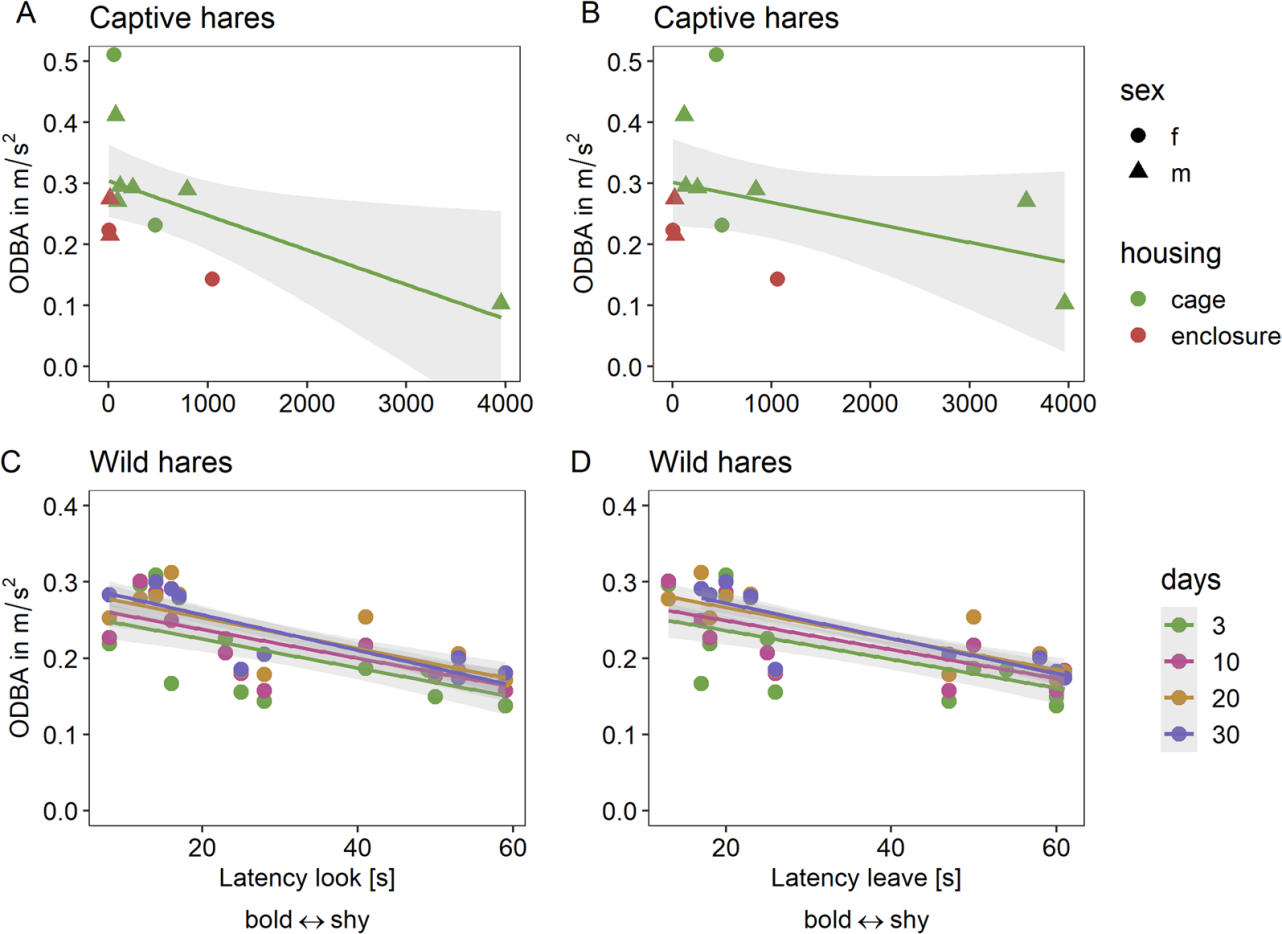
Observed values (circles and triangles), predicted values (connected by the black line), and SE (gray shading) for **A** The latency to first look out of a safe retreat; *p* = 0.003 and **B** the latency to leave a safe retreat in relation to general activity (mean ODBA measured in $\left[\frac{m}{{s}^{2}}\right]$) during the three consecutive days in a novel environment in 12 captive hares; *p* = 0.045. The behavioral trait **C** Latency-look and **D** Latency leave (small latencies equal to a high score on the bold-shy continuum) of wild hares (*Lepus europaeus*) and their relationship with general activity (mean ODBA) for the first 3 (n = 14), 10 (n = 13), 20 (n = 12) and 30 (n = 12) days of tracking; all *p* < 0.026

Consequently, we considered hares that took a comparatively long time to look out or leave the box as shy and hares that left the box quickly as bold individuals. The bolder an individual (i.e., the shorter Latency look and Latence leave), the higher the individual's activity (ODBA) during the first three successive days it roamed freely in the novel environment without effects of body mass (Fig. 2A, B, Table 2).

We found a similar pattern for free-ranging hares (single measurement of the latencies while releasing the individual). Hares that quickly looked out of the release box (Latency look) or left it (Latency leave) were also more active throughout the first 3, 10, 20 and 30 tracking days (Fig. 2C, D, Table 2).

### Personality effects on space use of wild hares

**Fig. 3:**
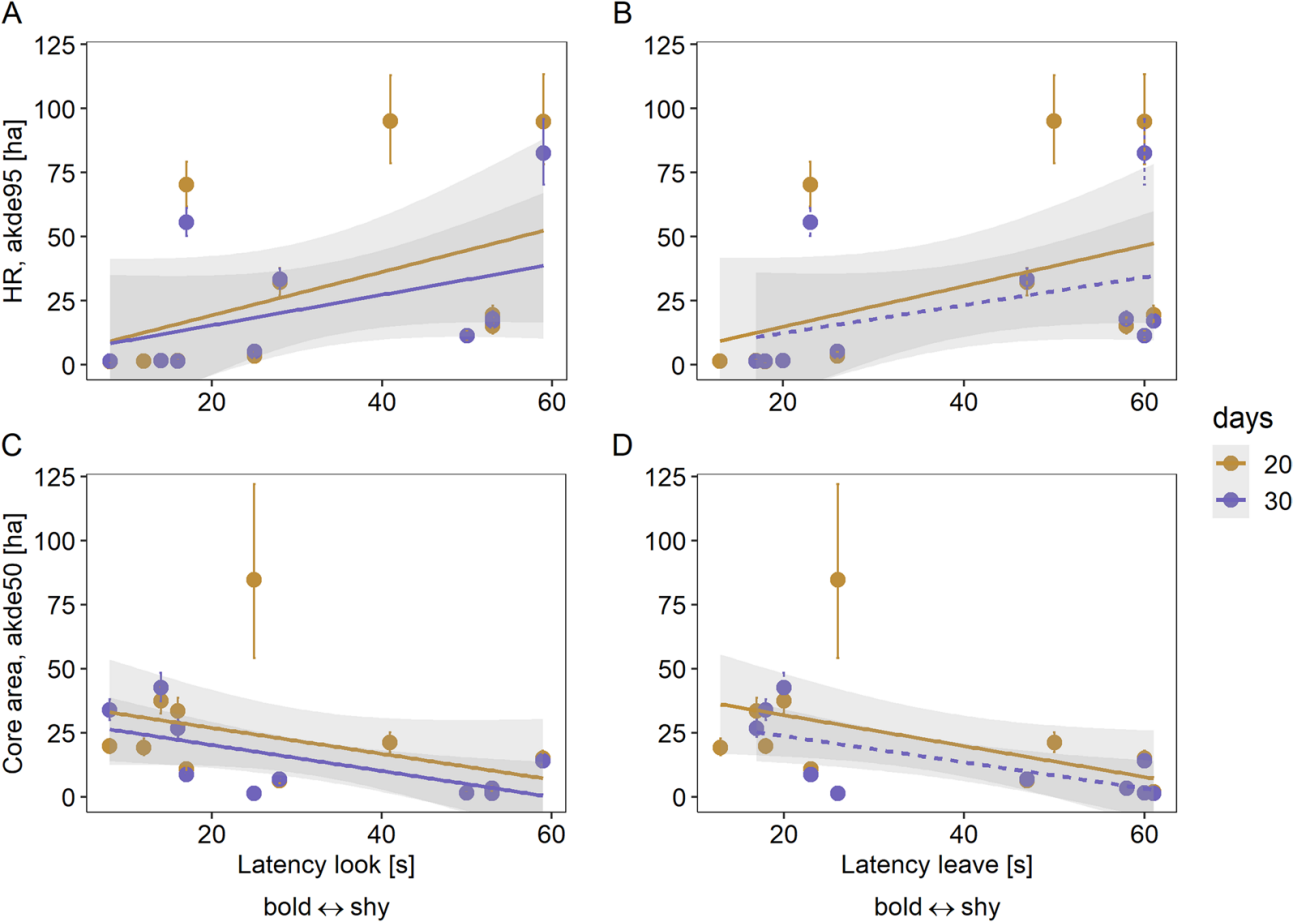
Observed values (circles) with low–high error bars (95% CI), predicted values (connected by lines, dashed lines show non-significant regressions), and SE (gray shading) of wild hares (*Lepus europaeus*) for relationships between behavioral variables **A** Latency look and **B** Latency leave with home range size (akde95) of the first 20- and 30 days after release; **C** Latency Look and **D** Latency leave with core area size (akde50) of the first 20- and 30 days after release. n = 12

Behavioral responses were shorter in wild hares (Latency look: 27 s ± 18 s and 26 s ± 18 s, Median ± SD; Latency leave: 37 ± 18 s and 26 s ± 19 s, Median ± SD) compared to captive hares (72 s ± 1017 s and 135 s ± 1276 s; Median ± SD). Bold behavioral types (i.e., individuals with fast behavioral respones) had smaller 20-day home ranges with larger 20-day core areas (Fig. 3, Table 3). The same pattern was found for 30 days and Latency look, whereas the link with Latency leave was almost marginally significant (Fig. 3, Table 3).

**Table 3:** Model results: home range sizes in relation to the Latency look and Latency leave^†^

Home range type	Latency look					Latency leave			
	Coefficients	Estimate	Std. error	t value	Pr( >|t|)	Estimate	Std. error	t value	Pr( >|t|)
20 days, akde50	(Intercept)	11.28	3.38	3.34	0.01	10.279	3.135	3.279	0.010
	Latency	− 0.04	0.01	− 2.86	0.02	− 0.041	0.012	− 3.310	0.009
	Mass	− 1.71	0.76	− 2.24	0.05	− 1.416	0.719	− 1.969	0.080
20 days, akde95	(Intercept)	− 2.130	5.578	− 0.382	0.711	− 0.622	5.336	− 0.117	0.910
	Latency	0.060	0.023	2.628	0.027	0.060	0.021	2.887	0.018
	Mass	0.579	1.259	0.460	0.656	0.145	1.224	0.118	0.909
30 days, akde50	(Intercept)	14.45	4.56	3.16	0.02	12.874	5.107	2.521	0.040
	Latency	− 0.04	0.02	− 2.57	0.04	− 0.034	0.017	− 2.003	0.085
	Mass	− 2.49	1.01	− 2.46	0.04	− 2.125	1.158	− 1.835	0.109
30 days, akde95	(Intercept)	− 8.590	5.948	− 1.444	0.192	− 6.547	6.193	− 1.057	0.326
	Latency	0.047	0.020	2.376	0.049	0.046	0.021	2.194	0.064
	Mass	2.076	1.320	1.573	0.160	1.567	1.404	1.116	0.301

†For our dependent variable, the logarithmic HR size, we assumed a Gaussian error distribution

## Discussion

By combining standardized behavioral tests under open field arena conditions and biologging of activity and space use of free-ranging individuals, we found that among-individual differences along the shy-bold continuum are consistent over time and related to overall activity and space use in European hares. Furthermore, we demonstrated how remote assessment of personality types without additional disturbance of the focal individual is achievable.

The variation in boldness of the hares was associated with short- and long-term differences in overall activity (ODBA) calculated from accelerometer measurements, with shy individuals having lower activity scores than bold individuals. Interestingly, this pattern was observed for both, hares under experimental conditions in an open field arena and hares in a wild population in an agricultural landscape in north-eastern Germany. In wild hares, heavier individuals were also less active. We can not exclude that this might also be partly related to an individual’s age, which was not determined during handling; however, all individuals were adults (> 3800 g, roughly above 2 years).

Notably, wild hares generally responded faster than captive hares. We suspect that this is due to captive hares being more used to handling and are therefore not as naive to humans as wild hares. Due to the relatively small sample sizes, we did not follow a covariance partitioning approach and did not account for differing habitat prerequisites (e.g., multivariate mixed models to study correlations between behavioral traits on various hierarchical levels [58]). Despite the limited sample size, the repeatability of behaviors compares well with findings from other taxa [e.g., meta-analysis: 91]. Further, we showed how behavioral traits covary with movement behavior leading to the dichotomy in space use patterns in a wild hare population. The positive correlation between general activity and boldness is consistent with previous findings using standardized behavioral tests, for example, in common voles, *Microtus arvalis* [73], bank voles *Myodes glareolus* [29], gray mouse lemurs, *Microcebus murinus* [74], and Siberian chipmunks, *Tamias sibiricus* [75]. This consistency highlights that the correlation also persists beyond short behavioral tests to longer-term and natural conditions. Our approach extends existing observations and shows that activity in relation to animal personality can be measured using animal-borne accelerometers in combination with simple novelty experiments.

Similarly, in eastern chipmunks, dawn activity and percentual activity per day (measured as ODBA via accelerometers) were positively related to exploration speed, although overall activity patterns varied according to temporal variability in food availability [22]. Behavioral variation along the shy-bold continuum could reduce intraspecific competition as individuals with varying levels of boldness likely also differ in their risk-taking or exploration behavior [30, 74]. Particularly in agricultural landscapes, bolder individuals may also be more resilient to disturbance from agricultural measures like soil tillage or harvest.

Boldness (and associated activity) was correlated with space use of hares in the wild population. Previous studies have shown that bolder individuals occupy more extensive home ranges, as found for bank voles (*Myodes glareolus*) [29], sleepy lizards (*Tiliqua rugosa*) [34], or common brushtail possums (*Trichosurus vulpecula*) [76]. Contrary to our expectation, bold hares had smaller home ranges during the initial 20 and 30 days after release, but with larger core areas.

Dissimilarity in space use and movement of bold and shy individuals might reflect different exploration styles between behavioral types along the shy-bold continuum. In general, resources are dispersed across a landscape [77, 78], but parts of a home range with higher resource density should be more important than those with fewer. Particularly bold individuals might successfully defend larger portions of these high-value areas, whereas shy individuals might be more likely to roam in search of less contested habitats, resulting in larger home ranges with smaller core areas. Hence, we suggest that bold hares are more successful in competing for valuable areas against shy individuals, forcing them to continue moving to find an unoccupied, suitable habitat.

This interpretation is tentatively supported by the energy expenditure of hares in our study, being negatively associated with body mass (non-significant trend only, Additional File 1: Fig. S2), which is consistent with findings of great tits *Parus major* [79] and Asian particolored bats *Vespertilio sinensis* [80]. Since bold animals tend to take more risks [81–83] and have higher energy costs, we suggest that occupying and defending a smaller area of higher forage quality might further allow bolder individuals to outweigh an increased risk of being detected by predators. This signisuggestion is in line with home range sizes varying largely among individuals. Although shy individuals in the present study presumably avoided risky situations, they occupied large home ranges with low habitat quality, i.e., they needed to move further to meet their energy demands and had lower feeding rates, possibly negatively affecting their fitness [81, 83, 84]. However, boldness has been shown to scale positively with (basal) metabolic rate in many species, and individuals with faster rates require more or higher-quality resources to meet their energetic demands [85–89]. Thus, for bold hares, dealing with risky situations and defending their home range against competitors could also be seen as a trade-off between energy expenditure versus habitat quality. Nevertheless, such behavioral variation might eventually facilitate the coexistence of individuals with varying behavioral phenotypes within the same population [5].

The link between movement ecology and animal personality is still in its infancy [2, 11], and the vast majority of studies on animal space use have been conducted without the inclusion of personalities, interpreting their variability mainly in terms of external factors or simple, measurable state variables, such as differences in sex or age. As wild animal populations are naturally composed of individuals differing in behavioral traits [17, 90], these inter-individual differences equip populations with a set of variable behavioral responses that could increase their resilience to fluctuating environmental conditions [53, 91, 92].

Considering animal personalities in space use studies might be crucial, as there is increasing evidence that sampling bias may inevitably influence the composition of animal personalities within a drawn sample and therefore the results of the respective study [4, 93]. Behavioral and ecological studies of various species may be affected, as the test subjects may not represent larger populations whose ecological patterns the researchers seek to understand. For example, due to well-established sampling protocols, bolder individuals are more likely to be trapped. In a study with pumpkinseed sunfish, *Lepomis gibbosus*, Wilson and others [94] noted that some fish were so shy that it was impossible to catch them even once, whereas bolder specimens were caught repeatedly [94]. Further studies have drawn attention to this personality-related sampling bias [95–99], suggesting that the assumption of random sampling might have been violated in many studies [100]. Although we do not know to what extent we could represent the extent of the shy-bold continuum in hares, we found substantial variation along its axis and demonstrated related differences in activity and space use.

## Conclusions

Carefully validated under standardized conditions, activity measurements via accelerometers, such as ODBA, could be a valuable tool to contribute to assessing behavioral types remotely. Considering that accelerometers are often already built into GPS units, we recommend activating them at least during the initial days of tracking to estimate individual variation in general activity and, if possible, match them with a simple novelty experiment. This additional information on individual behavioral types will help to explain variation in state-dependent behavior (e.g., risk-taking) and space use and further facilitate mechanistic understanding of processes that drive spatial and ecological dynamics in heterogeneous environments.

## Supplementary Information

**Additional file 1.** Contains supplementary figures and tables, including details of several of the methods used here, as well as model selection tables.

## Data Availability

GPS and acceleration data sets generated and analyzed during the current study are stored in the Movebank Data Repository (study IDs: 933567888, 1138520346). Together with the measured behavioral responses, these are available upon reasonable request from the corresponding author.
