# Supplementary material for: Personality drives activity and space use in a mammalian herbivore
Source: Mov Ecol. 2022 Aug 13;10:33. doi: 10.1186/s40462-022-00333-6 (PMC9375925; doi:10.1186/s40462-022-00333-6)
Supplement: Supplementary file 1 — Additional file 1. Contains supplementary figures and tables, including details of several of the methods used here, as well as model selection tables. [file 40462_2022_333_MOESM1_ESM.pdf]

Supplementary Information

Journal Title: **Movement Ecology**

Article Title: **Personality drives activity and space use in a mammalian herbivore**

Authors: **Jonas Stiegler, Alisa Lins, Melanie Dammhahn, Stephanie Kramer-Schadt, Sylvia Ortmann, Niels Blaum**

<u>**SI overview:**</u>

1) Home range size saturation (**Fig. S1**)

2) Activity and its relation to body mass (**Fig. S2**)

3) Tracking data overview (**Tab. S1**)

6) Model selection tables (**Tab S2, Tab S3**)

1) Home range size saturation

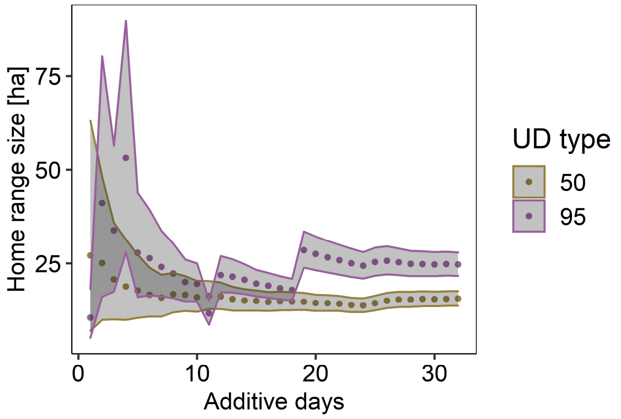

Figure S1: Home range sizes (akde95) and core ranges (akde50) for additive tracking days (1 - 32) of European hares (*Lepus europaeus*) in a disturbance-mediated landscape (Uckermark, Germany, 2020). Mean values (dots) and 95% confidence interval (grey filled area) for 10 individuals.

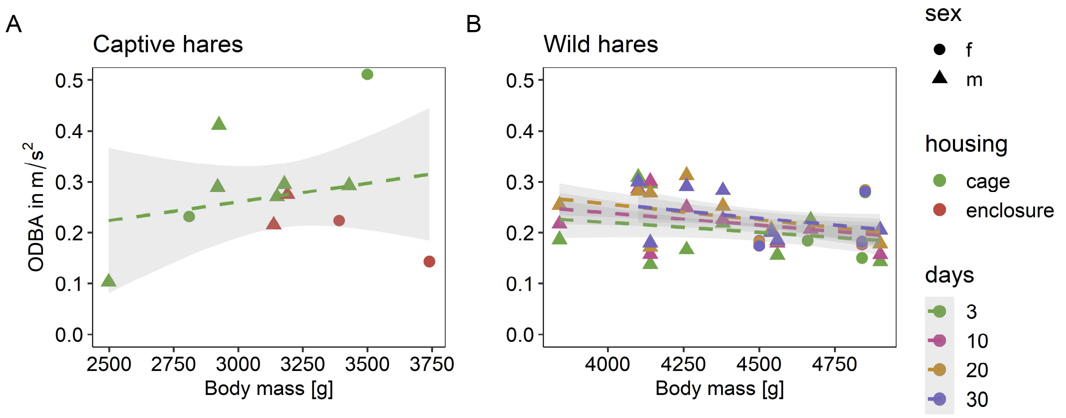

Figure S2: Activity and its relation to body mass of (A) captive hares and (B) wild hares. Measured values (dots and triangles) and non-significant regression lines for 3-days activity of captive and 3, 10, 20 and 30-days activity of wild hares. For sample size see Tab. S1)

2) Tracking data overview

Table S1: Overview of the tracking data and the respective sample size per analysis.

ID	*days tracked*	*body mass [g]*	*sex*	*analysis*	*n*
7427	4	4660	f	3-day activity	14
7398	19	4670	m	10-day activity	13
5581	20	4140	m	20-day activity	12
5559	22	3840	m	30-day activity	12
7393	32	4540	m	20-day HR (akde50)	12
7394	37	4100	m	20-day HR (akde95)	12
7396	54	4380	m	30-day HR (akde50)	12
7399	66	4850	f	30-day HR (akde95)	12
7400	72	4840	f		
7392	74	4900	m		
7397	77	4260	m		
7395	78	4140	m		
7391	95	4500	f		
7428	97	4560	m		

3) Model selection tables

Table S2: Model selection table of captive hares with BLUP derived from "Latency look ~ housing type + number of trial + (1|individual)", tracking data of 3 days, ranked by AICǂ

model	*(Int)*	*housing*	*BLUP*	*mass*	*df*	*logLik*	*AICc*	*delta*	*weight*
4	4.796	+	0.056		4	20.406	-27.097	0	0.512
3	4.018		0.062		3	17.848	-26.695	0.402	0.419
7	3.038		0.063	< 0.001	4	17.902	-22.09	5.007	0.042
8	5.793	+	0.055	< -0.001	5	20.482	-20.965	6.132	0.024
1	3.678				2	10.57	-15.806	11.292	0.002
2	4.666	+			3	11.628	-14.255	12.842	0.001
5	6.868			-0.001	3	10.881	-12.761	14.336	< 0.001
6	11.764	+		-0.002	4	13.133	-12.553	14.545	< 0.001

ǂ For our dependent variable, the mean ODBA of the first 3 tracking days of captive hares, we assume a Gamma distribution: $y_i \sim Gamma(\mu_i, v) with\ \mu_i = \exp(\beta_0 + \beta_1 \cdot mass_i + \beta_2 \cdot housing_i + \beta_3 \cdot BLUP_i)$. Here v is a dispersion parameter for which we have no particular interest.

Table S3: Model selection table of captive hares with BLUP derived from "Latency leave ~ housing type + number of trial + (1|individual)", tracking data of 3 days, ranked by AICǂ

model	*(Int)*	*housing*	*BLUP*	*mass*	*df*	*logLik*	*AICc*	*delta*	*weight*
3	3.804		0.097		3	14.089	-19.178	0	0.511
4	4.7	+	0.087		4	15.713	-17.711	1.467	0.245
1	3.678				2	10.57	-15.806	3.372	0.095
7	4.462		0.095	< -0.001	4	14.106	-14.497	4.681	0.049
2	4.666	+			3	11.628	-14.255	4.923	0.044
5	6.868			-0.001	3	10.881	-12.761	6.417	0.021
6	11.764	+		-0.002	4	13.133	-12.553	6.625	0.019
8	8.001	+	0.076	-0.001	5	16.191	-12.381	6.797	0.017

ǂ For our dependent variable, the mean ODBA of the first 3 tracking days of captive hares, we assume a Gamma distribution: $y_i \sim Gamma(\mu_i, v) with\ \mu_i = \exp(\beta_0 + \beta_1 \cdot mass_i + \beta_2 \cdot housing_i + \beta_3 \cdot BLUP_i)$. Here v is a dispersion parameter for which we have no particular interest.
